# *N*-Benzoyl-2-chloro­benzene­sulfonamide

**DOI:** 10.1107/S1600536810008731

**Published:** 2010-03-13

**Authors:** B. Thimme Gowda, Sabine Foro, P. A. Suchetan, Hartmut Fuess

**Affiliations:** a Department of Chemistry, Mangalore University, Mangalagangotri 574 199, Mangalore, India; b Institute of Materials Science, Darmstadt University of Technology, Petersenstrasse 23, D-64287 Darmstadt, Germany

## Abstract

In the crystal structure of the title compound, C_13_H_10_ClNO_3_S, the conformation of the of the N—H bond in the C—SO_2_—NH—C(O) segment is *anti* to the C=O bond. The dihedral angle between the two benzene rings is 73.3 (1)°. In the crystal, inversion dimers linked by pairs of N—H⋯O hydrogen bonds occur.

## Related literature

For background to our study of the effect of ring and side-chain substituents on the crystal structures of *N*-aromatic sulfonamides and for similar structures, see: Gowda *et al.* (2009 ▶; 2010 ▶); Suchetan *et al.* (2010 ▶).
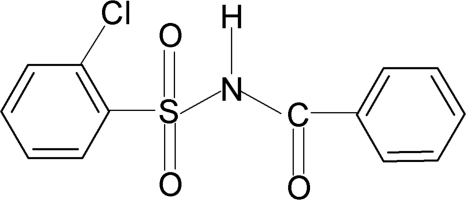


## Experimental

### 

#### Crystal data

C_13_H_10_ClNO_3_S
*M* *_r_* = 295.73
Triclinic, 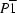

*a* = 8.1087 (8) Å
*b* = 9.3057 (9) Å
*c* = 9.6592 (9) Å
α = 74.841 (9)°
β = 65.790 (8)°
γ = 78.077 (9)°
*V* = 637.52 (11) Å^3^
*Z* = 2
Cu *K*α radiation
μ = 4.23 mm^−1^
*T* = 299 K
0.55 × 0.50 × 0.45 mm

#### Data collection

Enraf–Nonius CAD-4 diffractometer
3081 measured reflections
2210 independent reflections
2119 reflections with *I* > 2σ(*I*)
*R*_int_ = 0.026
3 standard reflections every 120 min  intensity decay: 1.0%

#### Refinement

*R*[*F* ^2^ > 2σ(*F* ^2^)] = 0.055
*wR*(*F* ^2^) = 0.147
*S* = 1.19
2210 reflections
176 parameters
1 restraint
H atoms treated by a mixture of independent and constrained refinement
Δρ_max_ = 0.68 e Å^−3^
Δρ_min_ = −0.66 e Å^−3^

### 

Data collection: *CAD-4-PC* (Enraf–Nonius, 1996 ▶); cell refinement: *CAD-4-PC*; data reduction: *REDU4* (Stoe & Cie, 1987 ▶); program(s) used to solve structure: *SHELXS97* (Sheldrick, 2008 ▶); program(s) used to refine structure: *SHELXL97* (Sheldrick, 2008 ▶); molecular graphics: *PLATON* (Spek, 2009 ▶); software used to prepare material for publication: *SHELXL97*.

## Supplementary Material

Crystal structure: contains datablocks I, global. DOI: 10.1107/S1600536810008731/ng2736sup1.cif

Structure factors: contains datablocks I. DOI: 10.1107/S1600536810008731/ng2736Isup2.hkl

Additional supplementary materials:  crystallographic information; 3D view; checkCIF report

## Figures and Tables

**Table 1:** Hydrogen-bond geometry (Å, °)

*D*—H⋯*A*	*D*—H	H⋯*A*	*D*⋯*A*	*D*—H⋯*A*
N1—H1*N*⋯O2^i^	0.85 (1)	2.12 (1)	2.968 (3)	172 (3)

Symmetry code: (i) 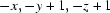.

**Fig. 1.**
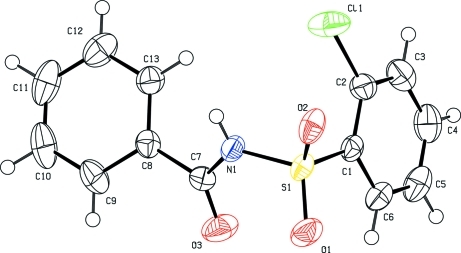
Molecular structure of the title compound, showing the atom-labelling scheme. Displacement ellipsoids are drawn at the 50% probability level.

**Fig. 2.**
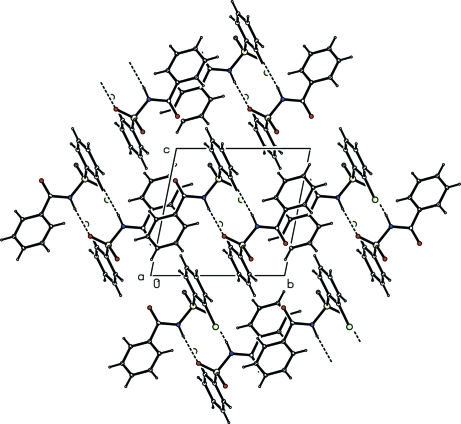
Molecular packing in the title compound. Hydrogen bonds are shown as dashed lines.

## supplementary crystallographic information

### Comment

Diaryl acylsulfonamides are known as potent antitumor agents against a broad spectrum of human tumor xenografts in nude mice. In the present work, as part of a study of the effect of ring and the side chain substituents on crystal structures of N-aromatic sulfonamides (Gowda et al., 2009; 2010; Suchetan et al., 2010),  the structure of N-(benzoyl)2-chlorobenzenesulfonamide (I) has been determined (Fig.1).

The conformation of the N—H bond in the C—SO_2_—NH—C(O) segment of the structure is anti to the C=O bond, similar to that observed in N-(benzoyl)benzenesulfonamide (II) (Gowda et al., 2009) and N-(2-chlorobenzoyl)benzenesulfonamide (III)(Gowda et al., 2010). The molecule is twisted at the s atom with a dihedral angle of 87.3 (1)° between the sulfonyl benzene ring and the C—SO_2_—NH—C—O segment, compared to the values of 86.5(0.1) in (II), and 87.3 (1)° (molecule 1) and 73.3 (1)° (molecule 2) in (III). Furthermore, the dihedral angle between the two benzene rings is 73.3 (1)° in (I) and 80.3(0.1) in (II), and 69.8 (1)° (molecule 1) and 89.8 (1)° (molecule 2) in (III).

The packing of molecules linked by intermolecular N—H···O (S) hydrogen bonds (Table 1) is shown in Fig. 2.

### Experimental

The title compound was prepared by refluxing a mixture of benzoic acid (0.02 mole), 2-chlorobenzenesulfonamide (0.02 mole) and excess phosphorous oxy chloride for 3 h on a water bath. The resultant mixture was cooled and poured into crushed ice. The solid, *N*-(benzoyl)2- chlorobenzenesulfonamide, obtained was filtered, washed thoroughly with water and then dissolved in sodium bicarbonate solution. The compound was later reprecipitated by acidifying the filtered solution with dilute HCl. The compound was filtered, dried and recrystallized.

Prism like colourless single crystals of the title compound used in X-ray diffraction studies were obtained by slow evaporation of its toluene solution at room temperature.

### Refinement

The H atom of the NH group was located in a difference map and later restrained to the distance N—H = 0.85 (1) Å. The other H atoms were positioned with idealized geometry using a riding model with C—H = 0.93 Å. All H atoms were refined with isotropic displacement parameters (set to 1.2 times of the U_eq_ of the parent atom).

### Crystal data

**Table:**

C_13_H_10_ClNO_3_S	*Z* = 2
*M**_r_* = 295.73	*F*(000) = 304
Triclinic, *P*1	*D*_x_ = 1.541 Mg m^−^^3^
Hall symbol: -P 1	Cu *K*α radiation, λ = 1.54180 Å
*a* = 8.1087 (8) Å	Cell parameters from 25 reflections
*b* = 9.3057 (9) Å	θ = 5.0–18.3°
*c* = 9.6592 (9) Å	µ = 4.23 mm^−^^1^
α = 74.841 (9)°	*T* = 299 K
β = 65.790 (8)°	Prism, colourless
γ = 78.077 (9)°	0.55 × 0.50 × 0.45 mm
*V* = 637.52 (11) Å^3^	

### Data collection

**Table:**

Enraf–Nonius CAD-4 diffractometer	*R*_int_ = 0.026
Radiation source: fine-focus sealed tube	θ_max_ = 66.9°, θ_min_ = 5.0°
graphite	*h* = −9→3
ω/2θ scans	*k* = −11→11
3081 measured reflections	*l* = −11→11
2210 independent reflections	3 standard reflections every 120 min
2119 reflections with *I* > 2σ(*I*)	intensity decay: 1.0%

### Refinement

**Table:**

Refinement on *F*^2^	Secondary atom site location: difference Fourier map
Least-squares matrix: full	Hydrogen site location: inferred from neighbouring sites
*R*[*F*^2^ > 2σ(*F*^2^)] = 0.055	H atoms treated by a mixture of independent and constrained refinement
*wR*(*F*^2^) = 0.147	*w* = 1/[σ^2^(*F*_o_^2^) + (0.0828*P*)^2^ + 0.435*P*] where *P* = (*F*_o_^2^ + 2*F*_c_^2^)/3
*S* = 1.19	(Δ/σ)_max_ = 0.004
2210 reflections	Δρ_max_ = 0.68 e Å^−^^3^
176 parameters	Δρ_min_ = −0.66 e Å^−^^3^
1 restraint	Extinction correction: *SHELXL97* (Sheldrick, 2008), Fc^*^=kFc[1+0.001xFc^2^λ^3^/sin(2θ)]^-1/4^
Primary atom site location: structure-invariant direct methods	Extinction coefficient: 0.034 (3)

### Special details

**Table:**

Geometry. All esds (except the esd in the dihedral angle between two l.s. planes) are estimated using the full covariance matrix. The cell esds are taken into account individually in the estimation of esds in distances, angles and torsion angles; correlations between esds in cell parameters are only used when they are defined by crystal symmetry. An approximate (isotropic) treatment of cell esds is used for estimating esds involving l.s. planes.
Refinement. Refinement of *F*^2^ against ALL reflections. The weighted *R*-factor wR and goodness of fit *S* are based on *F*^2^, conventional *R*-factors *R* are based on *F*, with *F* set to zero for negative *F*^2^. The threshold expression of *F*^2^ > σ(*F*^2^) is used only for calculating *R*-factors(gt) etc. and is not relevant to the choice of reflections for refinement. *R*-factors based on *F*^2^ are statistically about twice as large as those based on *F*, and *R*- factors based on ALL data will be even larger.

### Fractional atomic coordinates and isotropic or equivalent isotropic displacement parameters (Å^2^)

**Table:**

	*x*	*y*	*z*	*U*_iso_*/*U*_eq_	
Cl1	0.26198 (12)	0.56179 (10)	0.59662 (10)	0.0624 (4)	
S1	−0.08065 (8)	0.36244 (7)	0.76022 (7)	0.0312 (3)	
O1	−0.2217 (3)	0.2758 (3)	0.8694 (2)	0.0481 (6)	
O2	−0.1288 (3)	0.5111 (2)	0.6906 (2)	0.0433 (5)	
O3	0.0626 (3)	0.0405 (2)	0.7472 (3)	0.0534 (6)	
N1	0.0429 (3)	0.2791 (2)	0.6135 (2)	0.0332 (5)	
H1N	0.078 (4)	0.334 (3)	0.5232 (19)	0.040*	
C1	0.0729 (3)	0.3602 (3)	0.8476 (3)	0.0298 (6)	
C2	0.2197 (4)	0.4454 (3)	0.7766 (3)	0.0360 (6)	
C3	0.3387 (4)	0.4351 (4)	0.8484 (4)	0.0462 (8)	
H3	0.4368	0.4915	0.8012	0.055*	
C4	0.3125 (5)	0.3420 (4)	0.9892 (4)	0.0496 (8)	
H4	0.3929	0.3360	1.0374	0.059*	
C5	0.1694 (5)	0.2573 (4)	1.0606 (4)	0.0481 (8)	
H5	0.1529	0.1947	1.1566	0.058*	
C6	0.0499 (4)	0.2655 (3)	0.9892 (3)	0.0376 (6)	
H6	−0.0463	0.2072	1.0366	0.045*	
C7	0.1137 (3)	0.1292 (3)	0.6266 (3)	0.0323 (6)	
C8	0.2554 (4)	0.0906 (3)	0.4809 (3)	0.0319 (6)	
C9	0.2557 (4)	−0.0427 (3)	0.4419 (4)	0.0482 (8)	
H9	0.1671	−0.1059	0.5059	0.058*	
C10	0.3871 (6)	−0.0808 (4)	0.3085 (5)	0.0642 (11)	
H10	0.3846	−0.1683	0.2804	0.077*	
C11	0.5221 (5)	0.0087 (5)	0.2163 (4)	0.0620 (10)	
H11	0.6115	−0.0189	0.1267	0.074*	
C12	0.5257 (5)	0.1395 (4)	0.2561 (4)	0.0531 (8)	
H12	0.6191	0.1990	0.1946	0.064*	
C13	0.3913 (4)	0.1818 (3)	0.3865 (3)	0.0392 (7)	
H13	0.3915	0.2718	0.4115	0.047*	

### Atomic displacement parameters (Å^2^)

**Table:**

	*U*^11^	*U*^22^	*U*^33^	*U*^12^	*U*^13^	*U*^23^
Cl1	0.0634 (6)	0.0577 (6)	0.0523 (6)	−0.0258 (4)	−0.0168 (4)	0.0179 (4)
S1	0.0286 (4)	0.0371 (4)	0.0234 (4)	0.0013 (3)	−0.0053 (3)	−0.0100 (3)
O1	0.0312 (10)	0.0708 (15)	0.0354 (11)	−0.0156 (10)	−0.0013 (8)	−0.0106 (10)
O2	0.0486 (12)	0.0441 (12)	0.0323 (11)	0.0134 (9)	−0.0150 (9)	−0.0146 (9)
O3	0.0594 (14)	0.0387 (11)	0.0407 (12)	−0.0054 (10)	−0.0056 (10)	0.0050 (9)
N1	0.0396 (12)	0.0302 (11)	0.0245 (11)	−0.0018 (9)	−0.0072 (9)	−0.0068 (9)
C1	0.0296 (12)	0.0297 (12)	0.0261 (13)	0.0024 (10)	−0.0059 (10)	−0.0111 (10)
C2	0.0374 (14)	0.0289 (13)	0.0370 (15)	−0.0027 (10)	−0.0089 (11)	−0.0083 (11)
C3	0.0424 (16)	0.0448 (17)	0.057 (2)	−0.0060 (13)	−0.0188 (14)	−0.0184 (15)
C4	0.0502 (17)	0.0561 (19)	0.054 (2)	0.0074 (14)	−0.0298 (15)	−0.0235 (16)
C5	0.0548 (18)	0.0550 (19)	0.0315 (15)	0.0070 (15)	−0.0191 (14)	−0.0094 (14)
C6	0.0384 (14)	0.0398 (15)	0.0276 (14)	0.0008 (11)	−0.0078 (11)	−0.0067 (11)
C7	0.0353 (13)	0.0280 (12)	0.0329 (14)	−0.0057 (10)	−0.0124 (11)	−0.0038 (11)
C8	0.0364 (13)	0.0259 (12)	0.0342 (14)	0.0032 (10)	−0.0161 (11)	−0.0080 (10)
C9	0.0505 (17)	0.0339 (15)	0.066 (2)	0.0019 (13)	−0.0237 (16)	−0.0228 (15)
C10	0.070 (2)	0.058 (2)	0.081 (3)	0.0185 (18)	−0.036 (2)	−0.048 (2)
C11	0.055 (2)	0.077 (3)	0.049 (2)	0.0274 (19)	−0.0176 (16)	−0.0360 (19)
C12	0.0424 (17)	0.059 (2)	0.0432 (18)	0.0059 (14)	−0.0068 (14)	−0.0106 (15)
C13	0.0391 (14)	0.0345 (14)	0.0386 (16)	−0.0012 (11)	−0.0093 (12)	−0.0095 (12)

### Geometric parameters (Å, °)

**Table:**

Cl1—C2	1.730 (3)	C5—C6	1.382 (4)
S1—O1	1.421 (2)	C5—H5	0.9300
S1—O2	1.423 (2)	C6—H6	0.9300
S1—N1	1.644 (2)	C7—C8	1.482 (4)
S1—C1	1.762 (3)	C8—C13	1.387 (4)
O3—C7	1.206 (3)	C8—C9	1.387 (4)
N1—C7	1.388 (3)	C9—C10	1.370 (5)
N1—H1N	0.853 (10)	C9—H9	0.9300
C1—C6	1.382 (4)	C10—C11	1.369 (6)
C1—C2	1.398 (4)	C10—H10	0.9300
C2—C3	1.378 (4)	C11—C12	1.378 (5)
C3—C4	1.367 (5)	C11—H11	0.9300
C3—H3	0.9300	C12—C13	1.373 (4)
C4—C5	1.371 (5)	C12—H12	0.9300
C4—H4	0.9300	C13—H13	0.9300
			
O1—S1—O2	118.68 (13)	C1—C6—C5	120.2 (3)
O1—S1—N1	110.43 (12)	C1—C6—H6	119.9
O2—S1—N1	104.55 (11)	C5—C6—H6	119.9
O1—S1—C1	107.69 (13)	O3—C7—N1	121.8 (3)
O2—S1—C1	110.95 (12)	O3—C7—C8	124.1 (2)
N1—S1—C1	103.50 (12)	N1—C7—C8	114.1 (2)
C7—N1—S1	124.95 (19)	C13—C8—C9	119.5 (3)
C7—N1—H1N	117 (2)	C13—C8—C7	121.4 (2)
S1—N1—H1N	118 (2)	C9—C8—C7	119.0 (3)
C6—C1—C2	119.2 (3)	C10—C9—C8	119.7 (3)
C6—C1—S1	118.1 (2)	C10—C9—H9	120.2
C2—C1—S1	122.6 (2)	C8—C9—H9	120.2
C3—C2—C1	119.9 (3)	C11—C10—C9	120.7 (3)
C3—C2—Cl1	118.0 (2)	C11—C10—H10	119.7
C1—C2—Cl1	122.0 (2)	C9—C10—H10	119.7
C4—C3—C2	119.9 (3)	C10—C11—C12	120.1 (3)
C4—C3—H3	120.0	C10—C11—H11	120.0
C2—C3—H3	120.0	C12—C11—H11	120.0
C3—C4—C5	121.0 (3)	C13—C12—C11	119.9 (3)
C3—C4—H4	119.5	C13—C12—H12	120.0
C5—C4—H4	119.5	C11—C12—H12	120.0
C4—C5—C6	119.7 (3)	C12—C13—C8	120.1 (3)
C4—C5—H5	120.2	C12—C13—H13	120.0
C6—C5—H5	120.2	C8—C13—H13	120.0
			
O1—S1—N1—C7	−48.3 (3)	C2—C1—C6—C5	1.0 (4)
O2—S1—N1—C7	−177.1 (2)	S1—C1—C6—C5	178.5 (2)
C1—S1—N1—C7	66.7 (2)	C4—C5—C6—C1	−0.9 (4)
O1—S1—C1—C6	8.2 (2)	S1—N1—C7—O3	13.9 (4)
O2—S1—C1—C6	139.7 (2)	S1—N1—C7—C8	−165.87 (18)
N1—S1—C1—C6	−108.7 (2)	O3—C7—C8—C13	−136.3 (3)
O1—S1—C1—C2	−174.3 (2)	N1—C7—C8—C13	43.5 (3)
O2—S1—C1—C2	−42.9 (2)	O3—C7—C8—C9	41.1 (4)
N1—S1—C1—C2	68.7 (2)	N1—C7—C8—C9	−139.2 (3)
C6—C1—C2—C3	−0.4 (4)	C13—C8—C9—C10	−1.8 (5)
S1—C1—C2—C3	−177.8 (2)	C7—C8—C9—C10	−179.2 (3)
C6—C1—C2—Cl1	177.9 (2)	C8—C9—C10—C11	2.4 (5)
S1—C1—C2—Cl1	0.4 (3)	C9—C10—C11—C12	−0.8 (6)
C1—C2—C3—C4	−0.3 (4)	C10—C11—C12—C13	−1.5 (5)
Cl1—C2—C3—C4	−178.6 (2)	C11—C12—C13—C8	2.1 (5)
C2—C3—C4—C5	0.4 (5)	C9—C8—C13—C12	−0.5 (4)
C3—C4—C5—C6	0.2 (5)	C7—C8—C13—C12	176.9 (3)

### Hydrogen-bond geometry (Å, °)

**Table:**

*D*—H···*A*	*D*—H	H···*A*	*D*···*A*	*D*—H···*A*
N1—H1N···O2^i^	0.85 (1)	2.12 (1)	2.968 (3)	172 (3)

Symmetry codes: (i) −*x*, −*y*+1, −*z*+1.
